# Phantom and clinical evaluation of the effect of a new Bayesian penalized likelihood reconstruction algorithm (HYPER Iterative) on ^68^Ga-DOTA-NOC PET/CT image quality

**DOI:** 10.1186/s13550-022-00945-4

**Published:** 2022-12-12

**Authors:** Lei Xu, Can Cui, Rushuai Li, Rui Yang, Rencong Liu, Qingle Meng, Feng Wang

**Affiliations:** 1 Department of Nuclear Medicine, Nanjing First Hospital, Nanjing Medical University, Nanjing, 210006 Jiangsu China; 2 Department of PET/CT Center, Jiangsu Cancer Hospital, Jiangsu Institute of Cancer Research, The Affiliated Cancer Hospital of Nanjing Medical University, Nanjing, 210009 Jiangsu China

**Keywords:** PET, ^68^Ga-DOTA-NOC, Neuroendocrine neoplasm, Image reconstruction, Bayesian penalized likelihood, Penalization factor

## Abstract

**Background:**

Bayesian penalized likelihood (BPL) algorithm is an effective way to suppress noise in the process of positron emission tomography (PET) image reconstruction by incorporating a smooth penalty. The strength of the smooth penalty is controlled by the penalization factor. The aim was to investigate the impact of different penalization factors and acquisition times in a new BPL algorithm, HYPER Iterative, on the quality of ^68^Ga-DOTA-NOC PET/CT images. A phantom and 25 patients with neuroendocrine neoplasms who underwent ^68^Ga-DOTA-NOC PET/CT were included. The PET data were acquired in a list-mode with a digital PET/CT scanner and reconstructed by ordered subset expectation maximization (OSEM) and the HYPER Iterative algorithm with seven penalization factors between 0.03 and 0.5 for acquisitions of 2 and 3 min per bed position (m/b), both including time-of-flight and point of spread function recovery. The contrast recovery (CR), background variability (BV) and radioactivity concentration ratio (RCR) of the phantom; The SUV_mean_ and coefficient of variation (CV) of the liver; and the SUV_max_ of the lesions were measured. Image quality was rated by two radiologists using a five-point Likert scale.

**Results:**

The CR, BV, and RCR decreased with increasing penalization factors for four “hot” spheres, and the HYPER Iterative 2 m/b groups with penalization factors of 0.07 to 0.2 had equivalent CR and superior BV performance compared to the OSEM 3 m/b group. The liver SUV_mean_ values were approximately equal in all reconstruction groups (range 5.95–5.97), and the liver CVs of the HYPER Iterative 2 m/b and 3 m/b groups with the penalization factors of 0.1 to 0.2 were equivalent to those of the OSEM 3 m/b group (*p* = 0.113–0.711 and *p* = 0.079–0.287, respectively), while the lesion SUV_max_ significantly increased by 19–22% and 25%, respectively (all *p* < 0.001). The highest qualitative score was attained at a penalization factor of 0.2 for the HYPER Iterative 2 m/b group (3.20 ± 0.52) and 3 m/b group (3.70 ± 0.36); those scores were comparable to or greater than that of the OSEM 3 m/b group (3.09 ± 0.36, *p* = 0.388 and *p* < 0.001, respectively).

**Conclusions:**

The HYPER Iterative algorithm with a penalization factor of 0.2 resulted in higher lesion contrast and lower image noise than OSEM for ^68^Ga-DOTA-NOC PET/CT, allowing the same image quality to be achieved with less injected radioactivity and a shorter acquisition time.

**Supplementary Information:**

The online version contains supplementary material available at 10.1186/s13550-022-00945-4.

## Background

Positron emission tomography/computed tomography (PET/CT) imaging with gallium-68 (^68^ Ga)-DOTA-1-Nal3-octreotide (^68^Ga-DOTA-NOC) is increasingly used to image neuroendocrine neoplasms (NEN) due to its high accuracy in the detection, staging and assessment of the primary tumors or metastases and recurrence [1–4]. Generally, high image quality is essential to the precise interpretation of PET/CT clinical studies. The Bayesian penalized likelihood (BPL) reconstruction algorithm has been developed and clinically implemented to improve the image signal-to-noise ratio and lesion signal-to-background ratio compared to the widely used ordered subset expectation maximization (OSEM) algorithm for ^68^Ga tracers [5]; the superior performance of the BPL algorithm is due in part to its full iterative convergence without excessive noise amplification. Hence, the BPL algorithm has the potential to further improve quantitation accuracy [6], shorten acquisition time [7], and reduce the amount of radioactivity injected [8], while maintaining or even improving the image quality.

Recently, a new BPL algorithm, regularized expectation maximization image reconstruction (HYPER Iterative), was introduced by United Imaging Healthcare. HYPER Iterative incorporates the pixel-to-pixel total variation, global noise equivalent counts, and local sensitivity profile into the penalization term, in which the only user-adjustable parameter is the penalization factor that controls the trade-off between image noise level and resolution [9–11]. The Additional file 1 provides more details about the HYPER Iterative. Sui et al. showed that good image quality and diagnostic performance in total-body PET/CT can be ensured by the HYPER Iterative algorithm with ultralow 2-deoxy-2-[^18^F]-fluoro-d-glucose (^18^F-FDG) activity over a wide range of patient body mass indices [11]. For ^68^Ga tracers, previous studies by Liu et al. and Yang et al. showed that the HYPER Iterative algorithm provided significantly better lesion contrast and noise suppression than OSEM on PET/CT data captured with ^68^Ga-DOTA0-Tyr3-octreotate (^68^Ga-DOTA-TATE) or a ^68^Ga-labelled tracer targeting the prostate-specific membrane antigen (^68^Ga-PSMA) [12, 13]. However, no detailed analysis had been performed on the other radiopharmaceuticals and PET/CT scanners. Therefore, we conducted a phantom and patient study to investigate the impact of different penalization factors and acquisition durations for the HYPER Iterative algorithm on the quality of ^68^Ga-DOTA-NOC PET/CT images.

## Methods and materials

### Phantom data acquisition

A National Electrical Manufacturers Association (NEMA) image quality phantom was scanned on a SiPM-based digital time-of-fight PET/CT scanner (uMI780, United Imaging Healthcare). The PET scanner comprises a total of 101,920 LYSO crystals with dimensions of 2.76 × 2.59 × 118 mm^3^ and time-of-flight resolution of 520 ps. The four smallest spheres of the phantom (diameter = 10, 13, 17 and 22 mm) were filled with 13.2 kBq/mL of ^68^Ga solution; the concentration in these “hot” spheres was 4 times the background level. After 120 min of waiting time, the list-mode data were acquired.

The phantom data were acquired in list mode with an axial field of view of 30 mm. The images were reconstructed using the standard OSEM protocol recommended by the manufacturer (two iterations, 20 subsets, 3 mm Gaussian filter, time of flight, point-spread function model, scatter, CT-based attenuation and other necessary corrections) and HYPER Iterative (seven penalization factors: 0.03, 0.07, 0.1, 0.2, 0.3, 0.4, and 0.5) with 31-s and 46-s periods of list-mode data whose counts were comparable to the clinical acquisition protocols using 2 and 3 min per bed position (m/b). The reconstructed image gird was 192 × 192 and had a voxel size of 3.12 × 3.12 × 2.68 mm^3^. Thus, PET images were reconstructed in a total of 16 groups: O2 and O3 corresponded to OSEM with 2 and 3 m/b simulated data, respectively, while HR2.03, HR2.07, HR2.1, HR2.2, HR2.3, HR2.4, HR2.5, HR3.03, HR3.07, HR3.1, HR3.2, HR3.3, HR3.4, and HR3.5 corresponded to HYPER Iterative with 2 and 3 m/b and penalization factors ranging from 0.03 to 0.5, respectively.

### Phantom data evaluation

The reconstructed images were evaluated by percent contrast recovery (CR) and background variability (BV) for each sphere using the NEMA NU2-2012 image quality analysis tool (United Imaging Healthcare), as shown in Eqs. (1)–(2); the details can be found in the Additional file 1. The contrast-to-noise ratio (CNR) of the hot spheres was evaluated as the ratio of the contrast recovery to the background variability, which can be regarded as a measure of the signal level in the presence of noise. To compare the measurements to the true activity contrast, the radioactivity concentration ratio (RCR) was computed between the activity concentrations in the spheres and in the background. The radioactivity counts of each hot sphere were measured by placing a region of interest (ROI) on the sphere, matched to the sphere diameter; the standard deviation (SD) of phantom background counts was estimated by placing ROIs in the peripheral area of the phantom background in the slice passing through the centres of the spheres. Meanwhile, the normalized activity of each hot sphere was calculated as the mean activity concentrations of all reconstruction groups over that of O3, which revealed the relative change resulting from the different reconstructions using O3 as the reference.

1 $${\text{CR}}_{H,j} = (C_{H,j} /C_{B,j} - 1)/(a_{H} /a_{B} - 1) \times 100\%$$

2 $${\text{BV}}_{j} = {\text{SD}}_{j} /C_{B,j} \times 100\%$$

where CR_*H,j*_ is the percent CR of the sphere *j*; *C*_*H,j*_ and *C*_*B,j*_ are the average counts within an ROI on each sphere *j* and corresponding background ROIs; *a*_*H*_ and *a*_*B*_ are the activity concentrations in the sphere and the background of the phantom; BV_*j*_ is the percent BV measured in the background ROIs compared to sphere *j*; and SD_*j*_ is the standard deviation of the background ROI counts for sphere *j*.

### Patients

Twenty-five patients (ten men, fifteen women), who were admitted to Nanjing First Hospital between March 16 and June 16 of 2021 and underwent ^68^Ga-DOTA-NOC PET/CT imaging, were consecutively enrolled in this retrospective study. The inclusion criteria were as follows: the NEN was identified by pathology, and ^68^Ga-DOTA-NOC-avid lesions were found on PET images. Patients with visible liver metastases or unavailable raw data were excluded. The clinical study was approved by the ethics committee of Nanjing First Hospital, Nanjing Medical University (KY20171208-02) and performed in accordance with the principles of the Declaration of Helsinki and national regulations. Informed consent was waived due to the retrospective nature of this study. The mean age of the patients was 54.6 ± 12.2 years. The patients’ mean weight was 61.3 ± 9.7 kg, and their mean height was 1.63 ± 0.06 m. Seven patients were diagnosed with adrenal pheochromocytoma, three with paraganglioma, six with pancreatic NENs, four with lung NENs, three with rectal NENs, and two with retroperitoneal NENs. Further details regarding patient characteristics are listed in Table 1.

**Table 1:** Patient characteristics

Characteristics	Values
Sex	Male 10; Female 15
Age	54.6 ± 12.2 [35, 79] years
Height	1.63 ± 0.06 [1.55, 1.78] m
Weight	61.3 ± 9.7 [47.1, 80.0] kg
Body Mass index	22.9 ± 2.6 [18.3, 28.1] kg/m^2^
Uptake time	66.3 ± 15.3 [47, 97] minutes
Injected activity	97.2 ± 19.7 [55.7, 129.4] MBq
Injected activity per kilogram	1.6 ± 0.3 [1.0, 2.4] MBq/kg
Primary tumor	Pheochromocytoma (*n* = 7), Paraganglioma (*n* = 3), Pancreatic NET (*n* = 6), Lung NET (*n* = 4), Rectal NET (*n* = 3), Retroperitoneal NET (*n* = 2)

### Clinical image acquisition

The clinical acquisition protocol for ^68^Ga-DOTA-NOC PET/CT was with the same as the protocol described for the phantom study. The patients received 1.01–2.43 MBq/kg of ^68^Ga-DOTA-NOC and rested for approximately 66 min after administration (Table 1). PET/CT imaging was conducted from the skull base to the upper thigh in 3D list mode with an acquisition time of 3 m/b, and data were also reconstructed for 2 m/b. The PET image reconstruction settings and the naming rules for the 16 reconstruction groups were the same as in the phantom study.

### Quantitative evaluation of clinical images

The quality of the PET images was quantitatively assessed using the image noise level, which was defined as the percent coefficient of variation (CV) in the liver. A spherical volume of interest (VOI) with a diameter of 3 cm was first drawn on O3 in a site with uniform liver tissue, avoiding the vessels of the hepatic porta system, and this VOI was then copied and pasted to the other reconstruction groups; the mean and standard deviation of the standard uptake value (SUV_mean_ and SUV_sd_, respectively) were automatically measured in the VOI. The percent CV was calculated as SUV_sd_ over SUV_mean_. The normalized CV was defined as the ratio of CV for all groups to that for O3. Moreover, each lesion was delineated on PET images with a semi-automatic 3D segmentation tool by a nuclear radiologist; the maximum of standard uptake value (SUV_max_) and the volume of the lesion were then measured using 41% of SUV_max_ as the threshold [14]. SUV_max_ normalization was also performed in the same calculation as CV. The equivalent diameter (*D*) of the lesion was calculated as the diameter of a sphere with the same volume as the lesion.

### Qualitative assessment of clinical images

ThE PET images were independently evaluated on a dedicated workstation (uWS-MI R004, United Imaging Healthcare) by two nuclear radiologists with 10 years of experience each. All images were anonymized and labelled with randomly assigned numbers to reduce bias, and the radiologists rated the images without knowing the reconstruction settings. PET datasets were rated using a five-point Likert scale (1 = poor image quality with excessive noise or unnatural texture, and insufficient lesion depiction; 2 = unacceptable image quality with suboptimal noise, or poor lesion contrast and delineation leading to low diagnostic confidence; 3 = acceptable image quality with appropriate noise, sufficient lesion delineation, and sufficiently natural image texture to make a diagnosis; 4 = good image quality with optimal noise and satisfactory lesion delineation resulting in full diagnostic confidence. 5 = excellent image quality with almost zero noise, perfect contrast between the lesion and the background, and a sharp border delineating the lesion from the rest of the organ).

### Statistical analysis

GraphPad Prism 8 and Microsoft Excel 2016 were used for all statistical analyses. The data are presented as the mean ± SD. Since a precise measurement of true SUV was difficult to acquire in the patient study, the SUV of O3 was used as the reference for the comparisons between different reconstruction groups. A Paired t test was applied to compare the difference in lesion SUV_max_ between O3 and the other reconstruction groups if the data followed the normal distribution according to the D’Agostino–Pearson normality test. A matched-pairs Wilcoxon signed-rank test was used to examine the differences of liver CV and visual image quality scores between O3 and the other reconstruction groups. The *p* value was adjusted with the Benjamini–Hochberg correction to take into account the false discovery rate due to multiple comparisons. The inter-rater agreement of the visual image quality scores was measured by Cohen’s kappa test. In all analyses, *p* < 0.05 was considered to indicate statistical significance.

## Results

### Phantom study

**Fig. 1:**
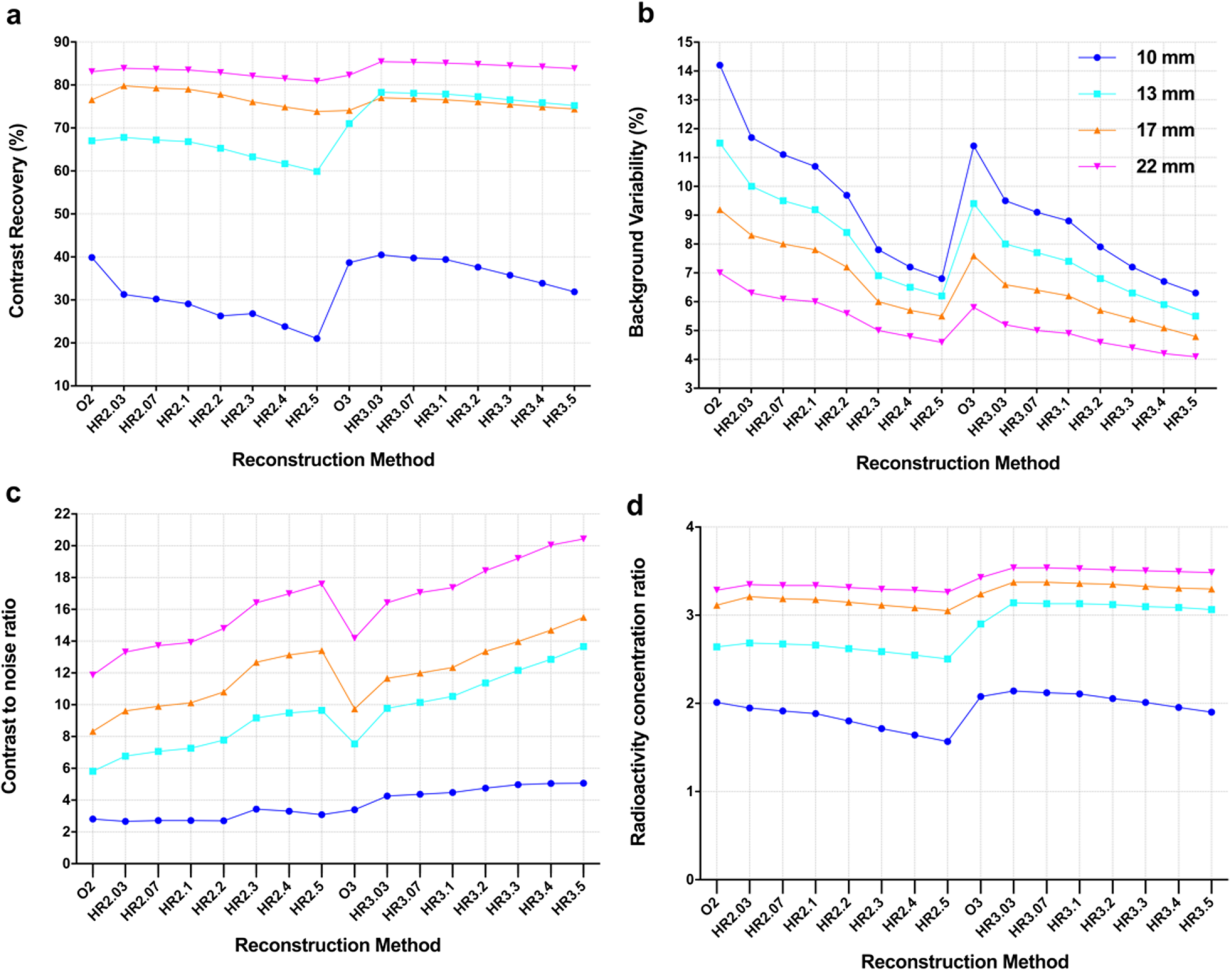
The plot of contrast recovery (**a**), background variability (**b**), contrast to noise ratio (**c**), and radioactivity concentration ratio (**d**) with different PET reconstruction methods for 4 hot spheres in the phantom

**Fig. 2:**
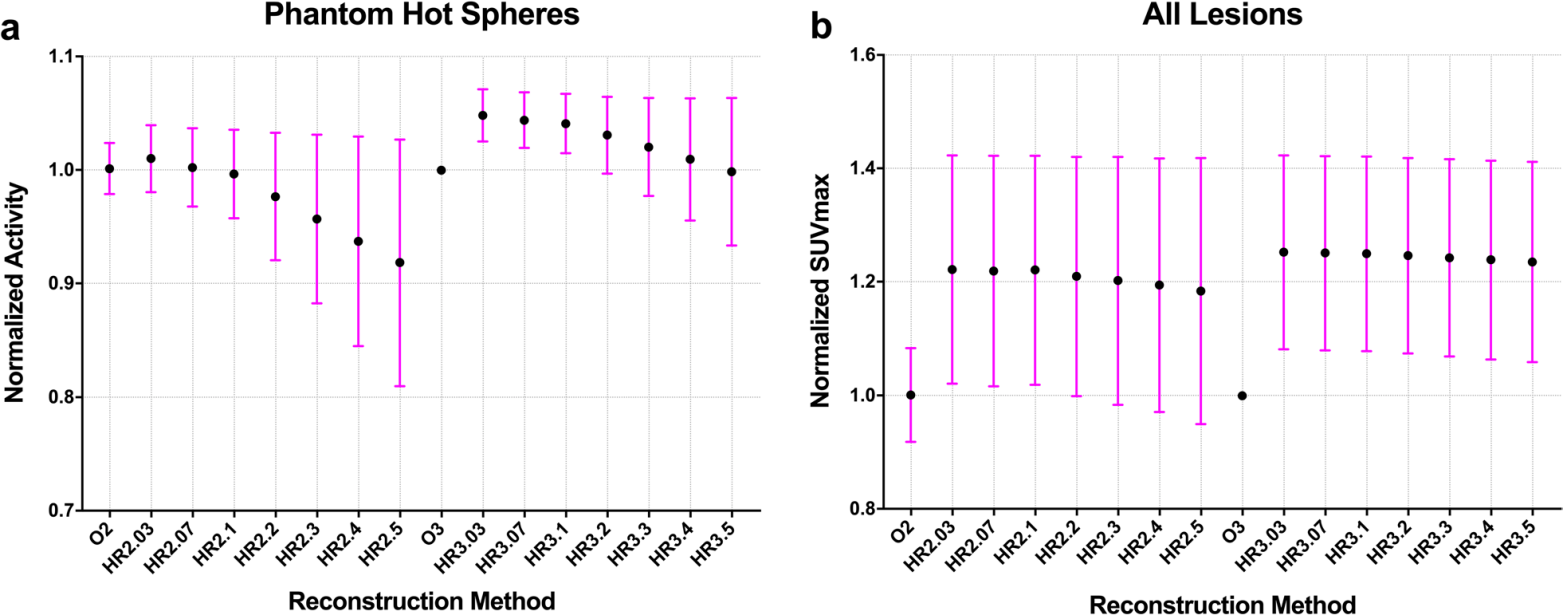
The relationship between normalized SUV_max_ and reconstruction methods. **a** The mean and SD of normalized activity change for the 4 hot spheres in the phantom study. **b** The mean and SD of normalized SUV_max_ for all lesions

The CRs of the four hot spheres decreased as the penalization factors increased except in the case of HR2.3, and a slight decline in the CR from HR3.03 to HR3.5 was observed in the three largest hot spheres, with diameters of 22 mm (85.4–83.8), 17 mm (77.0–74.4) and 13 mm (78.3–75.2) (Fig. 1a). The CRs of HR3.07 to HR3.1 were higher than those of O3 in all four hot spheres. For HR2.03 to HR2.5, the CRs were the highest for the 22 mm hot sphere, followed by 17 mm sphere and then the 13 mm sphere, reaching their lowest values for the 10 mm sphere. However, the CRs of the 13 mm hot sphere were slightly higher than those attained by the 17 mm hot sphere for HR3.03 to HR3.5 (Fig. 1a). Moreover, the mean of normalized activity was greater than 1.0 for HR2.03 to HR2.07(1.00–1.01), and HR3.03 to HR3.4 (1.01–1.05), very close to 1.0 for HR2.1 (0.996) and HR3.5 (0.998), and less than 1.0 for HR2.2 to HR2.5 (0.92–0.97) (Fig. 2a).

The BVs decreased with increasing penalization factors for each hot sphere. Moreover, the BVs of O2 were higher than those of O3 and the HYPER iterative 2 m/b and 3 m/b groups at the same diameters. The BVs were lowest for the 22-mm-diameter sphere, followed by 17- and 13-mm-diameter spheres, and highest for the 10-mm-diameter sphere with the same reconstruction method. The BVs of HR2.03 were higher than those of O3 at the same diameter, and the BVs of HR2.07 and HR2.1 were comparable to that of O3 because the difference was limited to a small range (− 1.0 to − 0.7%). Furthermore, other HYPER Iterative groups (HR2.2 to HR2.5 and HR3.03 to HR3.5) had more favorable BVs than O3 (Fig. 1b).

The CNRs of the four-hot spheres increased with increasing penalization factors. The CNRs of O3 were higher than those of O2. The CNRs declined with the diameter of hot spheres when the acquisition time was the same. Moreover, the CNRs of HR2.1 were equivalent to those of O3 except in 10 mm hot sphere (Fig. 1c). The RCRs of all reconstruction groups were less than the true contrast of 4. The RCRs slightly decreased with increasing penalization factors, acquisition time, and sphere diameter. The RCRs of O3 were lower than those of HR3.03 to HR3.1 at the same diameter, but higher than those of O2 and all HYPER Iterative groups with 2 min acquisition (Fig. 1d).

### Quantitative analysis of clinical study

The average liver SUV_mean_ was approximately equal in all reconstruction groups: 5.95 for O2, 5.96 for O3 and HR2.03 to HR2.5, and 5.97 for HR3.03 to HR3.5 (Table 2). The CVs of all reconstruction groups were less than 15%. In detail, the highest CV of the liver was 14.36 ± 3.38% and 12.88 ± 3.26% for O2 and O3, respectively (Table 2). The CV declined from 14.00 ± 3.00% to 10.59 ± 3.23% for HR2.03 to HR2.5, and from 13.52 ± 2.99% to 10.96 ± 3.11% for HR3.03 to HR3.5 (Table 2). The HR2.1, HR2.2, HR3.1, and HR3.2 groups were considered noise equivalent groups to O3 because their CVs did not make a significant difference (*p* = 0.113, 0.711, 0.079, and 0.287), and the image noise of HR2.03 and HR3.03 was equivalent to that of O2 (*p* = 0.525 and 0.055). The CVs of the HR2.03, HR2.07, HR3.03, and HR3.07 groups were significantly higher than those of O3 (all *p* < 0.01), whereas the CVs of HR2.3 to HR2.5 and HR3.3 to HR3.5 were significantly lower than those of O3 (all *p* < 0.01). Moreover, the mean normalized CV ranged from 0.83 to 1.12 for HR2.03-HR2.5 and 0.85 to 1.06 for HR3.03-HR3.5 (Table 2). The standard deviation of the normalized CV was lowest for HR3.03-HR3.1 (all 0.07), and increased with increasing penalization factor.

**Table 2:** SUV_mean_, CV of liver, and lesion SUV_max_, normalized lesion SUV_max_ of the clinical study

Group	SUV_mean_ of Liver	CV (%) of liver	Normalized CV	SUV_max_ of lesions	Normalized of SUV_max_
O2	5.95 ± 1.01	14.36 ± 3.38	1.12 ± 0.08	10.29 ± 6.14	1.00 ± 0.08
R2.03	5.96 ± 1.01	14.00 ± 3.00	1.10 ± 0.11	12.37 ± 6.95	1.22 ± 0.20
R2.07	5.96 ± 1.01	13.60 ± 2.98	1.07 ± 0.11	12.35 ± 6.96	1.22 ± 0.20
R2.1	5.96 ± 1.01	13.33 ± 3.03	1.05 ± 0.12	12.36 ± 6.95	1.22 ± 0.20
R2.2	5.96 ± 1.01	12.55 ± 3.08	0.98 ± 0.12	12.28 ± 7.00	1.19 ± 0.24
R2.3	5.96 ± 1.01	11.74 ± 3.11	0.92 ± 0.13	12.23 ± 7.03	1.20 ± 0.28
R2.4	5.96 ± 1.01	11.13 ± 3.16	0.87 ± 0.14	12.16 ± 7.05	1.19 ± 0.23
R2.5	5.96 ± 1.01	10.59 ± 3.23	0.83 ± 0.14	12.09 ± 7.10	1.18 ± 0.23
O3	5.96 ± 1.00	12.88 ± 3.26	1.00 ± 0.00	10.28 ± 6.01	1.00 ± 0.00
R3.03	5.97 ± 1.00	13.52 ± 2.99	1.06 ± 0.07	12.58 ± 6.71	1.25 ± 0.17
R3.07	5.97 ± 1.00	13.23 ± 2.98	1.04 ± 0.07	12.57 ± 6.71	1.25 ± 0.17
R3.1	5.97 ± 1.00	13.05 ± 3.00	1.02 ± 0.07	12.57 ± 6.71	1.25 ± 0.17
R3.2	5.97 ± 1.00	12.45 ± 2.98	0.97 ± 0.08	12.54 ± 6.72	1.25 ± 0.17
R3.3	5.97 ± 1.00	11.89 ± 3.01	0.93 ± 0.09	12.51 ± 6.73	1.24 ± 0.17
R3.4	5.97 ± 1.00	11.42 ± 3.05	0.89 ± 0.10	12.48 ± 6.74	1.24 ± 0.17
R3.5	5.97 ± 1.00	10.96 ± 3.11	0.85 ± 0.11	12.46 ± 6.76	1.23 ± 0.17

The lesion SUV_max_ decreased with increasing penalization factors except in the case of HR2.1 (Table 2). The lesion SUV_max_ of all HYPER Iterative 2 m/b and 3 m/b groups was significantly higher than that of O3 (all *p* < 0.001), and the lesion SUV_max_ of O2 (10.29 ± 6.14) was comparable to that of O3 (10.28 ± 6.01). The mean normalized SUV_max_ for the HYPER Iterative groups with 3 m/b was higher than those with 2 m/b when the penalization factor was the same. In detail, the mean lesion SUV_max_ increased 22–18% for HR2.03 to HR2.5, and 25–23% for HR3.03 to HR3.5 compared to O3 (Table 2 and Fig. 2b). We noted that the effect of the HYPER Iterative algorithm on the normalized activity seemed to be very different for the phantom than for the patients (Fig. 2a and b). This is due to the nonlinear total variation constraint in the HYPER Iterative algorithm, which may preserve hot spots with high contrast better than OSEM would do, and at the same time suppress hot spots with lower contrast more than OSEM would do.

The lesions were first divided into small (*D* < 10 mm, *n* = 13, range 7.7–9.9 mm), medium (10 ≤ 

**Fig. 3:**
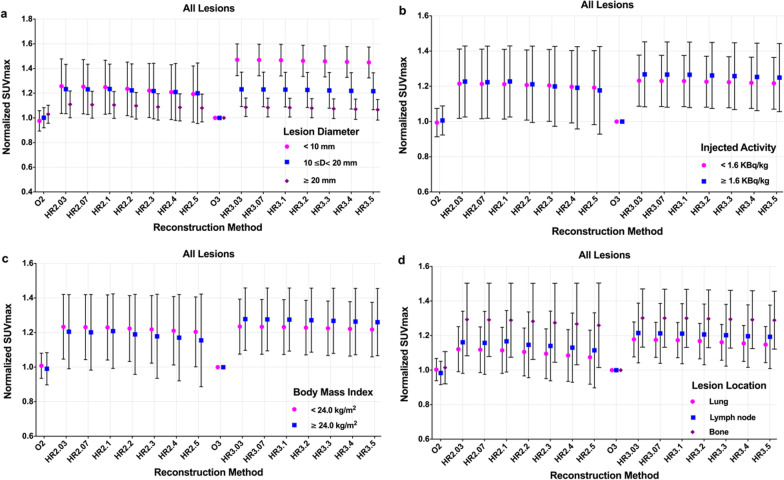
The mean and SD of normalized SUV_max_ for lesions divided by diameter (**a**), injected activity (**b**), patient body mass index (**c**), and location (**d**) with different reconstruction settings

*D* < 20 mm, *n* = 57, range 10.1–19.7 mm), and large (*D* ≥ 20 mm, *n* = 13, range 20.3–27.3 mm) categories according to their equivalent diameters. The mean normalized lesion SUV_max_ slightly decreased with the increased penalization factor for each category, and the mean normalized SUV_max_ for small lesions was higher than those for medium and large lesions across all HYPER Iterative 2 m/b and 3 m/b groups except for HR2.4 and HR2.5 at the same penalization factor and acquisition time (Fig. 3a). Minor changes in average normalized SUV_max_ were found for large lesions in all HYPER iterative groups, and minor changes were also found for the medium lesions. The mean normalized SUV_max_ was greater than 1.0 for large lesions (range 1.06–1.11) and 1.2 for medium lesions (range 1.20–1.23) in all HYPER Iterative groups. Meanwhile, the mean of normalized SUV_max_ for small lesions ranged from 1.19 to 1.25 in HR2.03-HR2.5 and increased to 1.45–1.47 in HR3.03-HR3.5.

The effect of the injected activity (IA) on the lesion SUV_max_ of different reconstruction procedures is demonstrated in Fig. 3b. The lesions were classified into low (IA < 1.60 MBq/kg, *n* = 35, range 1.01–1.59 MBq/kg) and high injected activity (IA ≥ 1.60 MBq/kg, *n* = 45, range 1.61–2.43 MBq/kg) according to our previous research and PET detection performance [12]. The mean normalized SUV_max_ for the high injected activity groups was slightly higher than that for the low injected activity groups in all HYPER Iterative groups except for HR2.3-H2.5 (Fig. 3b). Specifically, the mean normalized lesion SUV_max_ for all HYPER iterative groups was approximately 1.2 with a range of 1.19–1.23 for low activity and 1.18–1.27 for high activity. The mean of normalized SUV_max_ of HR3.03 to HR3.5 was comparable and slightly higher than that of HR2.03 to HR2.5 for low and high injected activity, respectively.

To investigate the effect of patient weight on the lesion SUV_max_, the patients were further sorted by body mass index (BMI) into two categories: an underweight to normal-weight group (BMI < 24 kg/m^2^, *n* = 47, range 18.36–23.72 kg/m^2^) and an overweight group (BMI ≥ 24 kg/m^2^, *n* = 33, range 24.22–28.12 kg/m^2^). The mean normalized lesion SUV_max_ of the underweight to normal-weight group was higher than that of the overweight group in the HYPER Iterative 2 m/b groups at the same penalization factor, while there was a trend in the opposite direction for 3 m/b acquisition. Additionally, the mean of normalized lesion SUV_max_ showed small changes between the two BMI categories in all HYPER iterative groups with the same penalization factor and acquisition time, but an obvious increase was found in the HYPER Iterative groups with 3 m/b acquisition compared to 2 m/b (Fig. 3c). The mean normalized lesion SUV_max_ was higher than 1.2 (range 1.20–1.23) and 1.15 (range 1.15–1.27) for the underweight to normal-weight group and overweight group, respectively, with HYPER Iterative.

A total of 80 ^68^Ga-DOTA-NOC-avid lesions were identified: eight lesions in the lung, 38 in the bone, two in the thyroid, 27 in the lymph nodes, four in the soft issue, and one in the pancreas. The mean normalized SUV_max_ was modestly decreased as the penalization factor increased for lung, lymph node and bone metastases. Moreover, the mean of the normalized SUV_max_ for the HYPER Iterative groups with 3 m/b acquisition was higher than that of the 2 m/b groups when the lesion location and the penalization factor were the same. Notably, the mean normalized lesion SUV_max_ was highest in the bone, second highest in the lymph node, and lowest in the lung at the same penalization factor. Furthermore, the mean normalized lesion SUV_max_ was greater than 1.0 (range 1.07–1.18) for the lungs, 1.1 (range 1.11–1.21) for lymph nodes, and 1.2 (range 1.26–1.30) for bones with HYPER Iterative, respectively (Fig. 3d).

### Qualitative comparison of clinical image quality

**Fig. 4:**
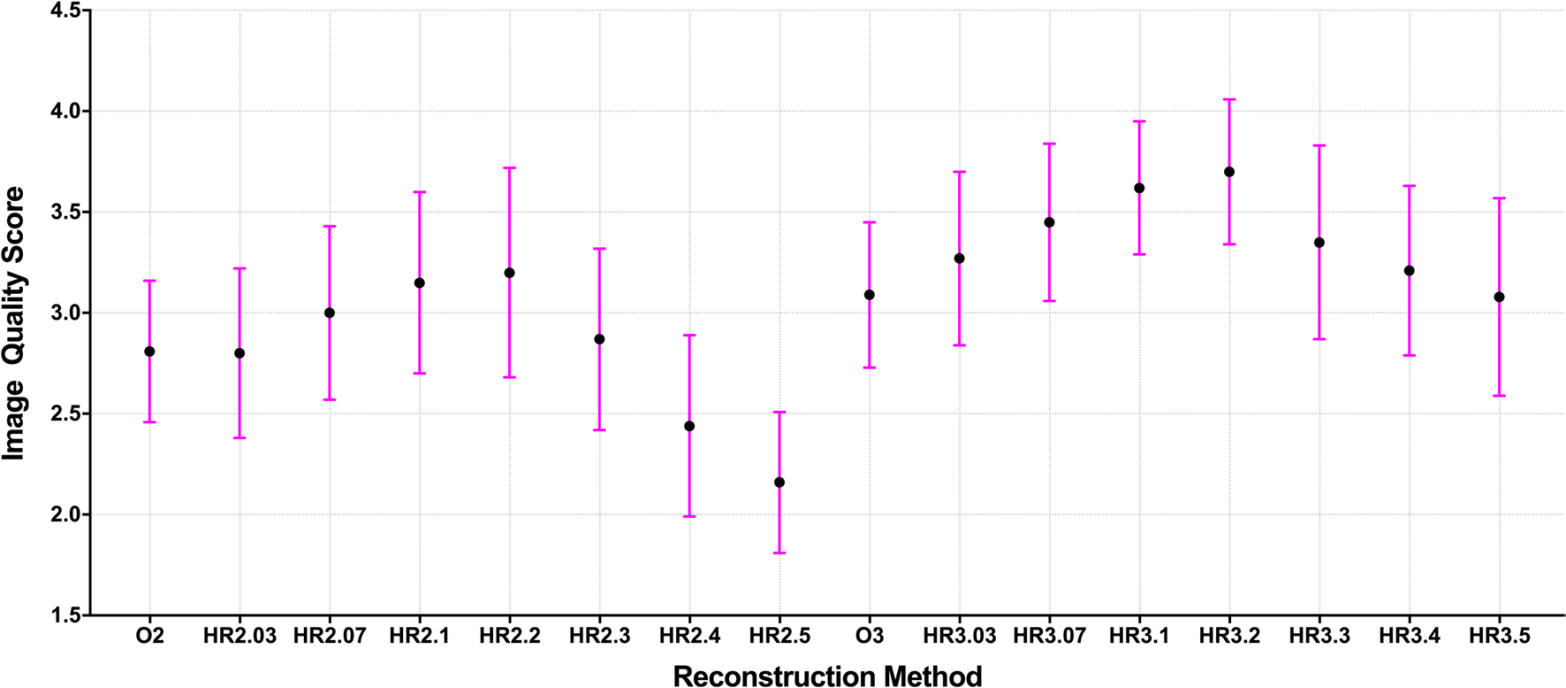
The qualitative image quality score of the clinical images with different reconstruction methods. The mean (filled circle) and standard deviation (error bar) of the image quality scores were plotted for each reconstruction method

**Fig. 5:**
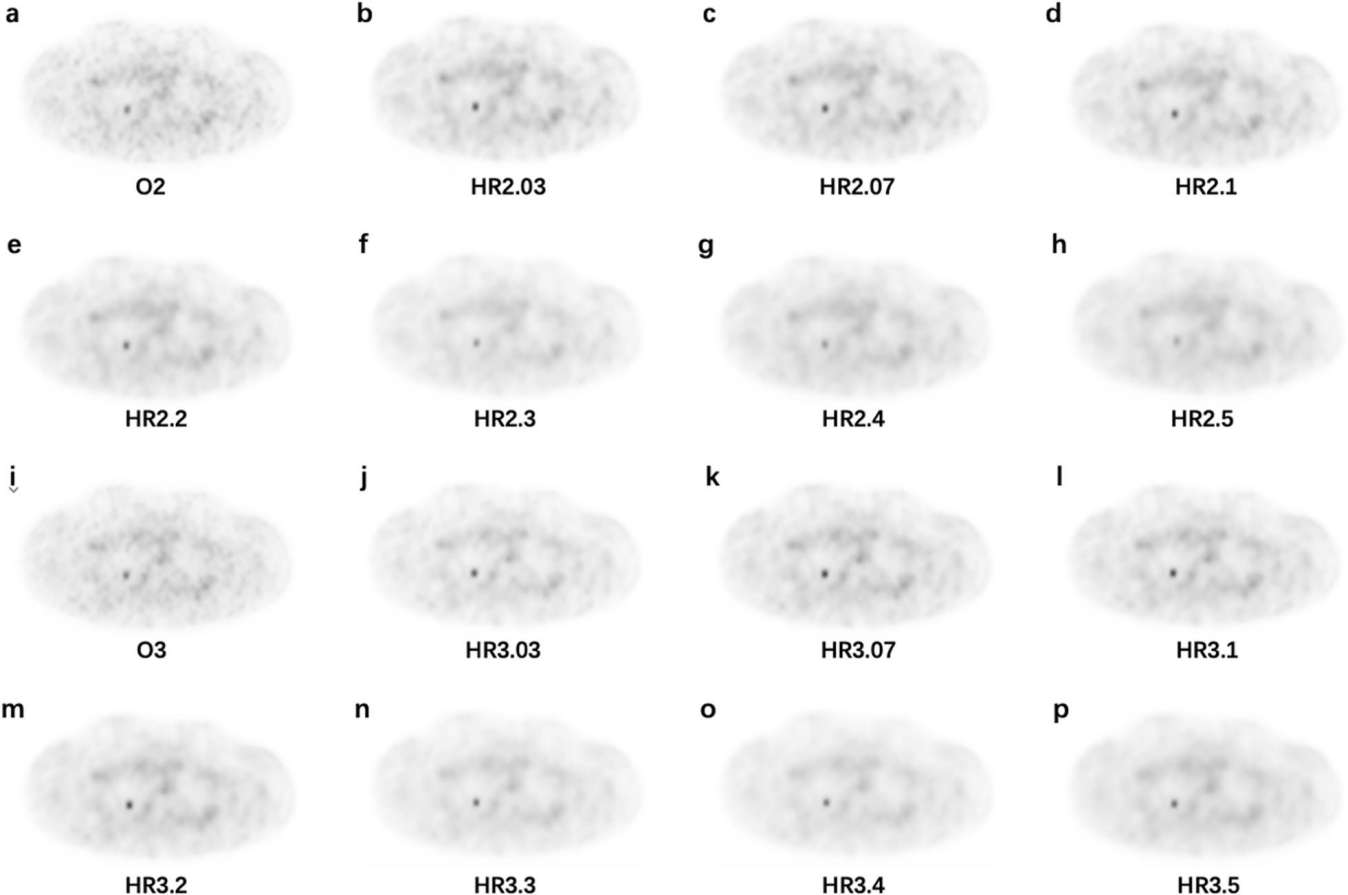
PET images of a 58-years-old female patient injected with 55.72 MBq ^68^Ga-DOTA-NOC diagnosed with adrenal pheochromocytoma (1.56 m, 55 kg, and resting for 73 min). A lung nodule with low DOTA-NOC uptake is depicted in the images. **a** O2, SUV_max_ = 2.23; **b** HR2.03, SUV_max_ = 2.99; **c** HR2.07, SUV_max_ = 2.88; **d** HR2.1, SUV_max_ = 2.80; **e** HR2.2, SUV_max_ = 2.49; **f** HR2.3, SUV_max_ = 2.16; **g** HR2.4, SUV_max_ = 1.8; **h** HR2.5, SUV_max_ = 1.46; **i** O3, SUV_max_ = 2.31; **j** HR3.03, SUV_max_ = 3.43; **k** HR3.07, SUV_max_ = 3.36; **l** HR3.1, SUV_max_ = 3.37; **m** HR3.2, SUV_max_ = 3.18; **n** HR3.3, SUV_max_ = 3.01; **o** HR3.4, SUV_max_ = 2.81; **p** HR3.5, SUV_max_ = 2.57

**Fig. 6:**
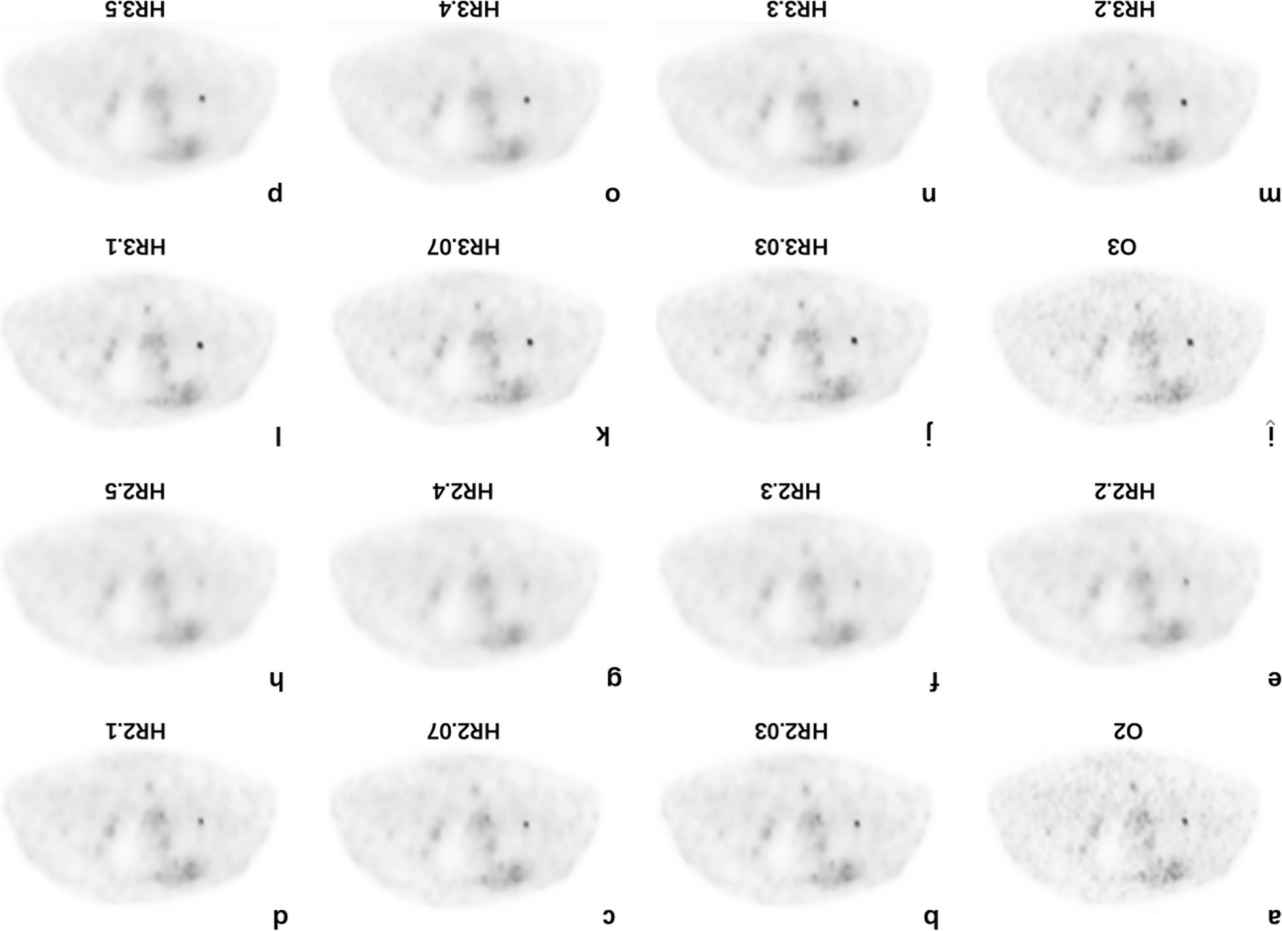
A 69-years-old male patient injected 119.88 MBq ^68^Ga-DOTA-NOC diagnosed with pancreatic NEN (1.70 m, 60 kg, and resting for 89 min). The images demonstrate a small bone lesion with a diameter of 0.88 cm measured on CT image (not shown) and low DOTA-NOC uptake. **a** O2, SUV_max_ = 3.82; **b** HR2.03, SUV_max_ = 3.57; **c** HR2.07, SUV_max_ = 3.30; **d** HR2.1, SUV_max_ = 3.07; **e** HR2.2, SUV_max_ = 2.21; **f** HR2.3, SUV_max_ = 1.50; **g** HR2.4, SUV_max_ = 1.16; **h** HR2.5, SUV_max_ = 1.05; **i** O3, SUV_max_ = 4.47; **j** HR3.03, SUV_max_ = 4.72; **k** HR3.07, SUV_max_ = 4.64; **l** HR3.1, SUV_max_ = 4.60; **m** HR3.2, SUV_max_ = 4.41; **n** HR3.3, SUV_max_ = 4.17; **o** HR3.4, SUV_max_ = 3.85; **p** HR3.5, SUV_max_ = 3.44

**Fig. 7:**
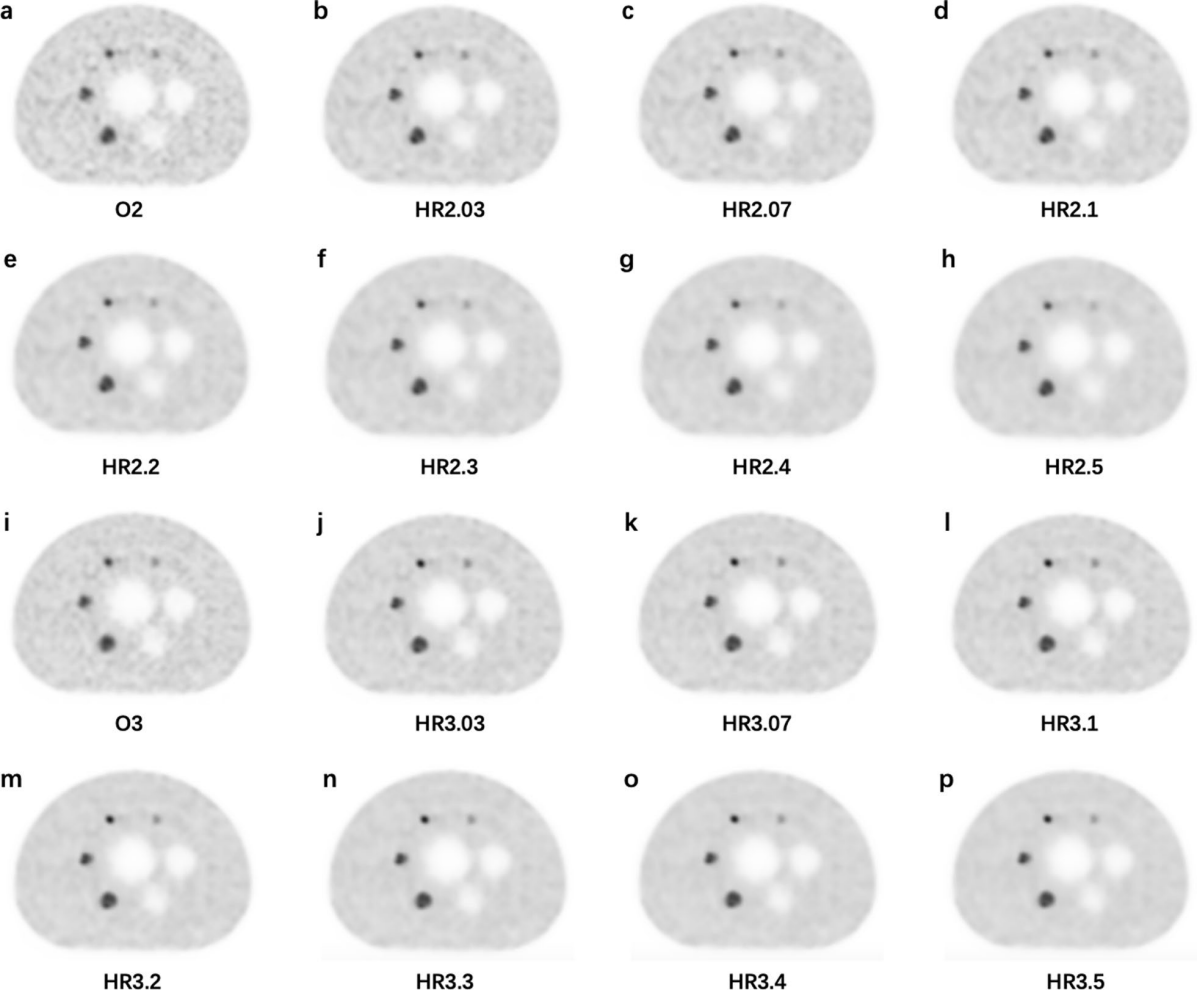
The images for the NEMA phantom with 4 hot spheres filled with ^68^Ga-DOTA-NOC in a 4:1 contrast ratio. **a** O2, **b** HR2.03, **c** HR2.07, **d** HR2.1, **e** HR2.2, **f** HR2.3, **g** HR2.4, **h** HR2.5, **i** O3, **j** HR3.03, **k** HR3.07, **l** HR3.1, **m** HR3.2, **n** HR3.3, **o** HR3.4, **p** HR3.5

**Fig. 8:**
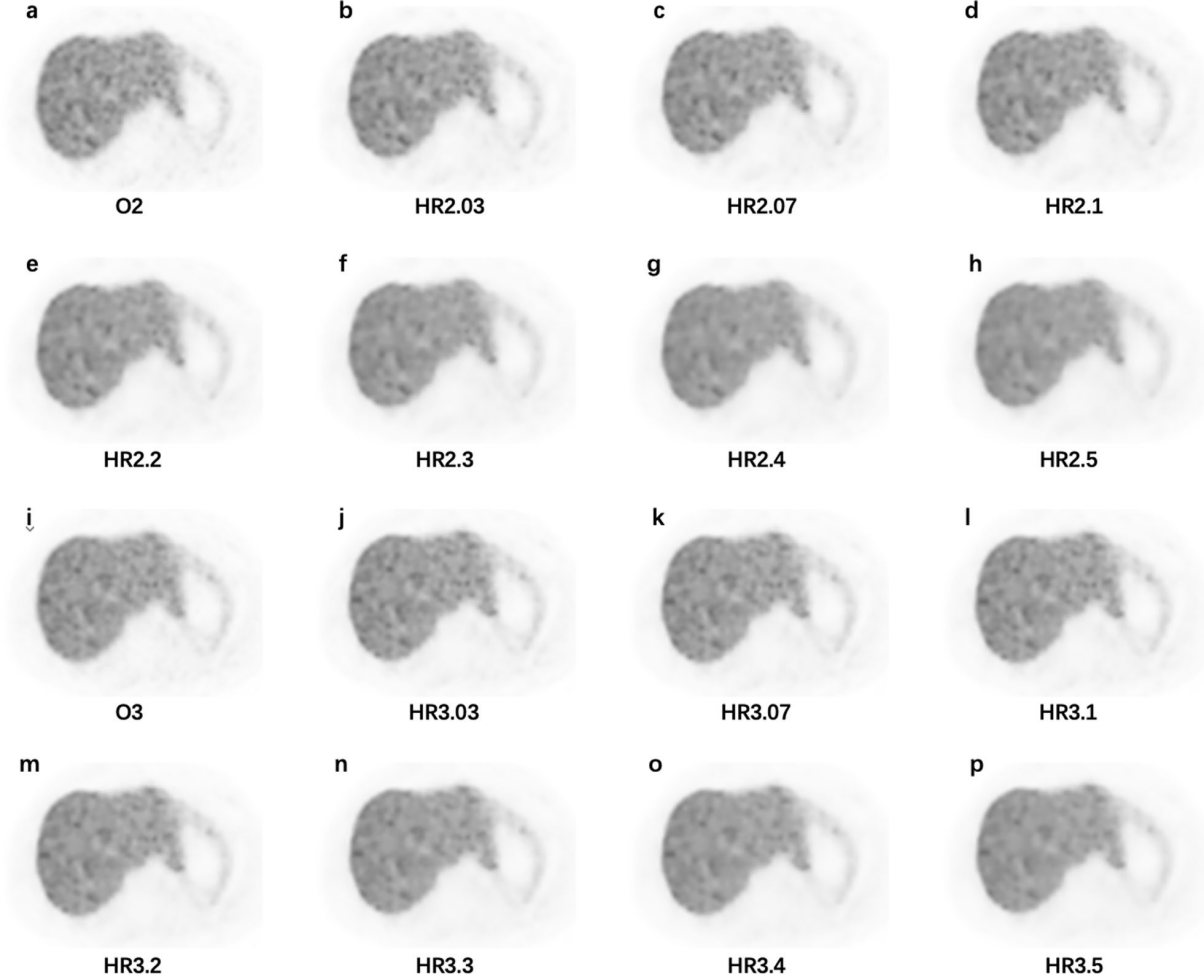
Axial PET images in the liver from a 45-years-old male patient injected 101 MBq ^68^Ga-DOTA-NOC with adrenal pheochromocytoma for the different reconstruction series (1.68 m, 75 kg, and resting for 50 min). **a** O2, CV = 13.45; **b** HR2.03, CV = 12.73; **c** HR2.07, CV = 12.23; **d** HR2.1, CV = 11.89; **e** HR2.2, CV = 10.91; **f** HR2.3, CV = 10.07; **g** HR2.4, CV = 9.41; **h** HR2.5, CV = 8.74; **i** O3, CV = 11.24; **j** HR3.03, CV = 11.28; **k** HR3.07, CV = 11.11; **l** HR3.1, CV = 10.94; **m** HR3.2, CV = 10.27; **n** HR3.3, CV = 9.76; **o** HR3.4, CV = 9.27; **p** HR3.5, CV = 8.77

**Fig. 9:**
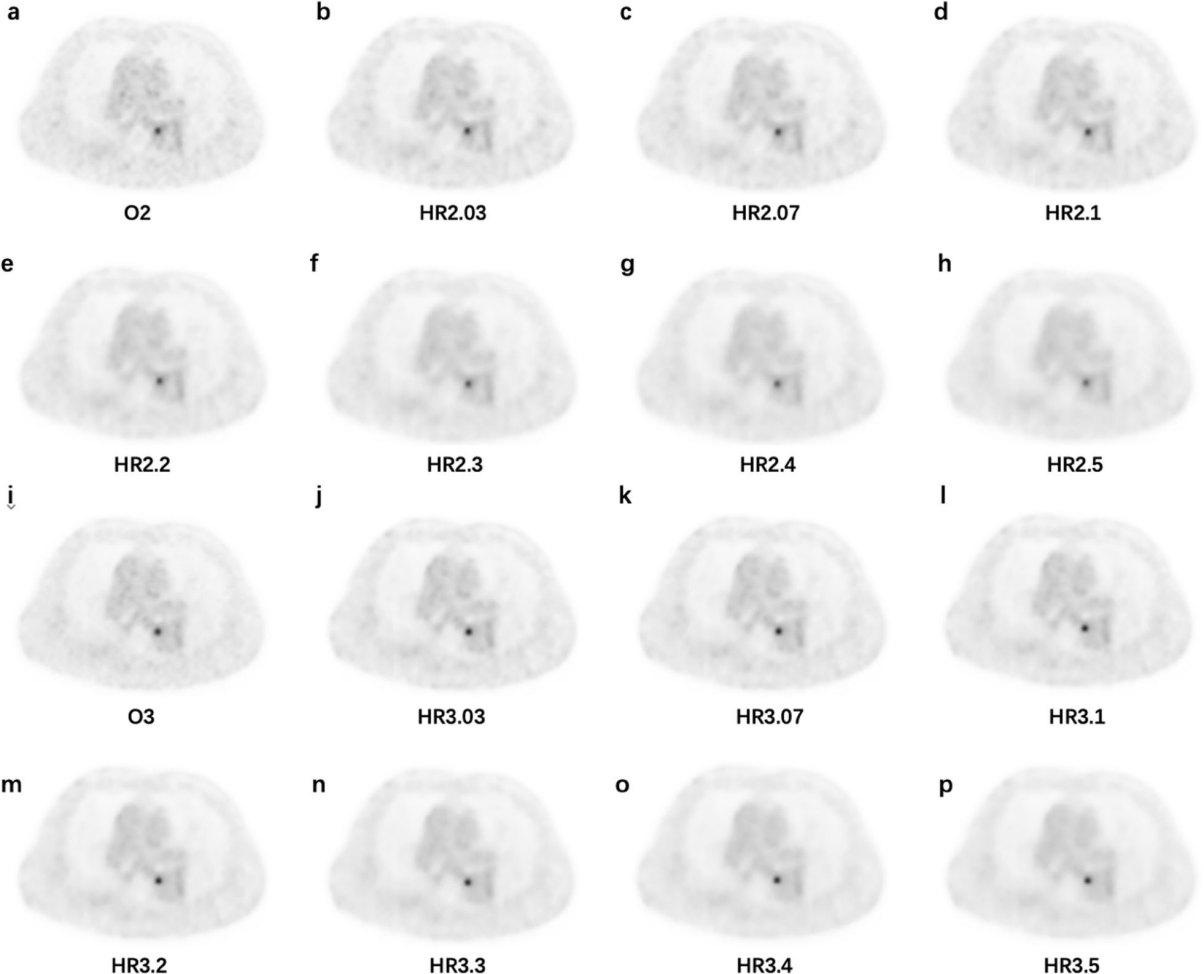
A 79-years-old male patient injected 115.81 MBq ^68^Ga-DOTA-NOC diagnosed with adrenal pheochromocytoma (1.65 m, 70 kg, and resting for 80 min). The images reveal a lymph node with a diameter of 1.13 cm measured on CT image (not shown) and moderate DOTA-NOC uptake. **a** O2, SUV_max_ = 6.17; **b** HR2.03, SUV_max_ = 6.23; **c** HR2.07, SUV_max_ = 6.11; **d** HR2.1, SUV_max_ = 6.02; **e** HR2.2, SUV_max_ = 5.7; **f** HR2.3, SUV_max_ = 5.35; **g** HR2.4, SUV_max_ = 4.90; **h** HR2.5, SUV_max_ = 4.61; **i** O3, SUV_max_ = 6.49; **j** HR3.03, SUV_max_ = 7.11; **k** HR3.07, SUV_max_ = 7.07; **l** HR3.1, SUV_max_ = 7.04; **m** HR3.2, SUV_max_ = 6.93; **n** HR3.3, SUV_max_ = 6.82; **o** HR3.4, SUV_max_ = 6.7; **p** HR3.5, SUV_max_ = 6.58

The mean image quality score first increased and then declined with increasing penalization factors (Fig. 4). The highest image quality score was assigned to HR2.2 (3.20 ± 0.52) and HR3.1 (3.70 ± 0.36) for the 2 m/b and 3 m/b acquisition groups. The lowest score was acquired at HR2.4 (2.44 ± 0.45) and HR2.5 (2.16 ± 0.35) due to poor contrast for small lesions (Figs. 5 and 6), and the second lowest score was given to O2 (2.81 ± 0.35) because of poor image noise (Figs. 7, 8, and 9). The average scores of HR3.07 to HR3.3 were significantly higher than that of O3 (all *p* < 0.05), and the image quality scores of HR2.07 to HR2.3 did not differ from that of O3 (all *p* > 0.062). The inter-rater agreement was substantial (*k* = 0.71).

## Discussion

We investigated the effect of the HYPER Iterative algorithm on the quality of ^68^Ga-DOTA-NOC PET/CT images and focused on determining the optimal penalization factor using the phantom and patient data. The HYPER Iterative 2 m/b groups with a penalization factor between 0.07 and 0.2 could offer a 19–22% increase in lesion SUV_max_ and a 0–10% reduction in noise compared to the OSEM 3 m/b group while reducing the acquisition time by one-third. Our qualitative and quantitative results suggested that a penalization factor of 0.2 could provide the optimal image quality for ^68^Ga-DOTA-NOC PET/CT with lower image noise and higher lesion contrast for different lesion sizes, patient weights, amount of injected activity, and tumor locations.

Inappropriate selection of the penalization factor may cause overestimation or underestimation of noise, and results in over- or under-smoothed images [11–13]. The choice of an optimal penalization factor was challenging and usually affected by the radiopharmaceutical, the acquisition setting, the radiologists’ preference, small difference in the BPL algorithms, and measures of image quality. Therefore, the optimal penalization factor is often given as a reference range. The phantom and clinical studies concluded that a penalty factor of 0.8–0.9 was optimal for detecting small tumors on ^18^F-FDG PET using small voxels and HYPER Iterative reconstruction [14]. Another study on total-body PET reconstruction with ultra-low ^18^F-FDG activity showed that good image quality and diagnostic performance could be ensured by the HYPER Iterative algorithm with a penalty factor of 0.3–0.5 in obese patients [11]. For ^68^Ga tracers, one preliminary ^68^Ga-DOTA-TATE PET/CT study concluded that the HYPER Iterative algorithm at 2 m/b with a penalization factor of 0.21 or at 3 m/b with a penalization factor of 0.35 resulted in the highest image quality, and the range of recommended penalization factors for clinical practice was 0.14 to 0.35 [12]. However, another study indicated that the highest image quality for ^68^Ga-PSMA PET was achieved with the HYPER Iterative algorithm at 3 m/b with a penalization factor of 0.14, and the optimal penalization factor was between 0.14 and 0.21 [13]. In line with those studies, our study suggested that a penalization factor between 0.03 and 0.5 for HYPER Iterative reconstruction provided higher lesion contrast compared to OSEM, where the lowest image noise was achieved between 0.1 and 0.5. Moreover, both HYPER Iterative 2 m/b and 3 m/b acquisitions with a penalization factor between 0.1 and 0.2 could attain equivalent image noise to an OSEM 3 m/b acquisition. Therefore, the recommended penalization factors should always be chosen according to the radiopharmaceutical, the acquisition settings and the criteria for the optimal image quality, as these factors change the choice of the penalization factor.

Our data implied that the SUV_max_ of the large lesions (diameter > 20 mm) increased more than 6% in all HYPER Iterative 2 m/b and 3 m/b groups compared to the OSEM 3 m/b group, and a 20% increase was found for medium lesions (10 mm ≤ diameter < 20 mm), while the SUV_max_ of the small lesions (diameter < 10 mm) increased more than 19% for all HYPER Iterative 2 m/b groups, and the increase was up to 45% for 3 m/b groups. These results were in accordance with the findings of a previous study using ^68^Ga-PSMA PET/CT in which the contrast of the large lesions (diameter > 20 mm) increased 10% in the HYPER Iterative algorithm with a penalization factor of 0.14 compared to OSEM, while the increase was 20% for small lesions (diameter < 20 mm) [13]. Another study using ^18^F-FDG PET/CT found that the conspicuity and SUV_max_ of lung lesions < 10 mm in diameter were significantly higher on images reconstructed by the BPL algorithm than by OSEM [15], but the present study did not find significant difference between BPL and OSEM for lesions > 10 mm in diameter. Therefore, our results supported the hypothesis that the HYPER Iterative algorithm could improve the contrast of small lesions and improve conspicuity regardless of lesion size.

By current procedure guidelines, the recommended activity of for ^68^Ga-DOTA-conjugated peptides ranges from 100 to 300 MBq depending on the PET imaging characteristics [16, 17]. Our data showed that, regardless of the injected activity per kilogram, HYPER Iterative had a higher lesion SUV_max_ than OSEM, but the penalization factor had a limited influence on the increase in lesion SUV_max_ between HYPER Iterative groups with the same acquisition time, and the lesion SUV_max_ increased slightly as acquisition time was increased from 2 m/b to 3 m/b. These results were in accordance with a previous study that evaluated the influence of different penalization factors for different activity-time products in whole-body ^18^F-FDG PET/CT [18]. Notably, the average injected activity in our study was less than 100 MBq, which may potentially reduce the patient dose and acquisition time.

An increase in patient weight may cause increasing noise and consequently affect the quality of PET images [19]. A previous study of ^18^F-FDG showed that the BPL algorithm provided a more consistent liver signal-to-noise ratio than OSEM with increasing patient BMI [20]. Our study demonstrated that with increasing penalization factors, the gap in gains of lesion SUV_max_ was minimized and became relatively stable in the two BMI groups for 2 m/b and 3 m/b acquisition. Moreover, the gains in lesion SUV_max_ were nearly equivalent for normal to underweight patients with increasing penalization factor, but the relative difference between the 2 m/b and 3 m/b groups was larger for overweight patients with the same penalization factor, which means that patients with greater weight can benefit much more from the HYPER Iterative reconstruction with longer acquisition.

No previous studies have been performed on the impact of the BPL algorithm on PET imaging performance according to the lesion location. Our study found that the gain of lesion SUV_max_ was highest for bone, second for the lymph nodes, and lowest for the lungs with the same penalization factor, but the gains of SUV_max_ were higher for lungs and lymph nodes between 2 m/b and 3 m/b acquisition, which may result from tumor uptake and patient characteristics.

A shorter acquisition time is important for patient comfort and throughput in any busy clinical setting. Previous studies have shown that the BPL algorithm is able to shorten the acquisition time in ^18^F-FDG and ^68^Ga tracer PET/CT imaging [21–23]. Our results indicated that the HYPER Iterative algorithm could reduce the duration of ^68^Ga-DOTA-NOC PET by one-third with equivalent or improved image quality compared to 3 m/b OSEM. However, further studies will be needed to explore the potential of the BPL algorithm for improving the conspicuity of malignant tumors and maintaining the image quality for delayed PET imaging.

The results of qualitative image quality ratings by nuclear radiologists depend on several factors: the personal experience, clinical tasks, image noise and contrast. In our study, the highest average quality score was given to the penalization factor of 0.2 for both 2 m/b and 3 m/b acquisition. The selection penalization factor was equal to or greater than that of the groups with equivalent noise (HR2.1 to HR2.2 and HR3.1 to HR3.2). However, the coefficients of variation of all the HYPER Iterative groups were less than the recommended maximal tolerance (15%) in our clinical practice. The raters often preferred the images with a lower background noise level or higher tumor-to-background contrast ratio because these images could promote diagnostic confidence for the detection of all malignancies, especially for small and low-contrast lesions. We also noted that the image noise level was less than 13% when the image quality score was highest at a penalization factor of 0.2. In addition, further increasing the penalization factor might produce smoother images but might also result in less contrast enhancement, indicating that this noise level should be considered as the target noise setting to achieve high image quality in practical ^68^Ga-DOTA-NOC PET/CT. This phenomenon was consistent with the previous studies using the BPL algorithm with a high penalization factor for ^68^Ga tracer PET [12, 13].

In clinical practice, the penalization factor should be fixed to maintain the consistency of SUVs. In a previous study of ^68^Ga-DOTA-TATE PET, the optimal penalization factor was determined from the equivalent noise group with increased tumor SUV_max_ and improved signal-to-background ratio [24]. The factor also depended on the lesion detection rate and patient throughput in oncologic whole-body ^18^F-FDG examinations [18]. Since radiologists focus on diagnosis as the primary task and their preference may largely be based on their experience with OSEM, it is appropriate to consider the raters’ experience with the BPL reconstruction algorithm. Therefore, our study recommends a penalization factor of 0.2 for 2 m/b and 3 m/b acquisition as the optimal choice based on comprehensive analysis, providing a fine balance between visual assessment and quantitative evaluation.

Last but not least, when a PET study is performed for follow-up during treatment, it becomes more important to use a standardized acquisition and reconstruction protocol, ensuring that the scan duration and reconstruction parameters are identical, and that the amount of injected activity is very similar for all PET scans acquired during follow-up. Therefore, it would be prudent to use the HYPER Iterative instead of OSEM in the follow-up studies because SUV measurements differ with different reconstruction algorithms.

Our study has several limitations. First, only 25 patients were included in this work due to the time-consuming task of the image reconstruction with different penalization factors. A larger number of suspected or untreated NEN patients should be involved in future studies. Second, the relationship between SUV measurement under HYPER Iterative reconstruction and pathological results needs to be investigated; such information could improve the early differential diagnosis of NENs. Furthermore, the noise-smoothing ability of the HYPER Iterative algorithm could be applied to dynamic PET imaging or late-phase imaging for lesion detection.

## Conclusions

When applied to ^68^Ga-DOTA-NOC PET/CT data, HYPER Iterative reconstruction algorithm with a penalization factor of 0.2 can improve lesion contrast as well as reduce image noise compared to OSEM, enabling a shortened acquisition time and a reduction in injected activity while maintaining the image quality.

## Supplementary Information

**Additional file 1.** Phantom evaluation.

## Data Availability

The datasets used and analyzed during the current study are available from the corresponding author on reasonable request.

## Abbreviations

^68^Ga-DOTA-NOC: Gallium-68-DOTA-1-Nal3-octreotide
PET: PET/CT: positron emission tomography/computed tomography
NEN: Neuroendocrine neoplasms
BPL: Bayesian penalized likelihood
OSEM: Ordered subset expectation maximization
^18^F-FDG: 2-Deoxy-2-[^18^F]-fluoro-d-glucose
NEMA: National Electrical Manufacturers Association
CR: Contrast recovery
BV: Background variability
CNR: Contrast-to-noise ratio
RCR: Radioactivity concentration ratio
ROI: Region of interest
VOI: Volume of interest
SD: Standard deviation
SUV: Standard uptake value
HR: HYPER Iterative
*D*: Equivalent diameter
CV: Coefficient of variation
^68^Ga-PSAM: ^68^Gallium-labelled tracer targeting the prostate-specific membrane antigen
^68^Ga-DOTA-TATE: ^68^Gallium-DOTA0-Tyr3-octreotate
