# Supplementary material for: Phantom and clinical evaluation of the effect of a new Bayesian penalized likelihood reconstruction algorithm (HYPER Iterative) on 68Ga-DOTA-NOC PET/CT image quality
Source: EJNMMI Res. 2022 Dec 12;12:73. doi: 10.1186/s13550-022-00945-4 (PMC9742075; doi:10.1186/s13550-022-00945-4)
Supplement: Supplementary file 1 — Additional file 1. Phantom evaluation. [file 13550_2022_945_MOESM1_ESM.docx]

**Additional file 1**

**Section 1. HYPER Iterative**

The base of the HYPER Iterative algorithm can be written as the following objective function [1,2]:

$$\hat{f}={argmax}_{f\geq0}\left[ \sum_{ij} -p_{ij}f_{j}+\sum_{i} c_{i}\ln\left( \sum_{j} p_{ij}f_{j} \right)-\sum_{j} \gamma_{j}\times U\left( f_{j} \right) \right] \left( 1 \right)$$

$$\gamma_{j}=g\left( NEC, {snns}_{j} \right)\times\beta\left( 2 \right)$$

$$U\left( f \right)=\sum_{x,y,z} \left| \nabla f \right| \left( 3 \right)$$

Where f is the estimated image. i and j are the indexes of the projection bins and the image pixels, respectively. c_i_ are the measured emission data. p_ij_ is the system matrix indicating the counts emitted from the jth image pixel detected by the ith projection bin. $\gamma_{j}$ is a parameter of regularized strength. U is the total variation penalization of the pixels in the neighborhood. sns_j_ is the spatially varied sensitivity profile. NEC presents the noise equivalent counts. g is a function of NEC and sns_j_. $\beta$ is a factor representing penalty strength which is normalized to a range of 0 to 1.0.

[1] Sui X, Tan H, Yu H, et al. Exploration of the total-body PET/CT reconstruction protocol with ultra-low ^18^F-FDG activity over a wide range of patient body mass indices. *EJNMMI Phys*. 2022;9(1):17.

[2] Xu L, Li RS, Wu RZ, et al. Small lesion depiction and quantification accuracy of oncological ^18^F-FDG PET/CT with small voxel and Bayesian penalized likelihood reconstruction. *EJNMMI Phys*. 2022;9(1):23.

**Section 2.**

The percent contrast recovery (CR) and background variability (BV) for each sphere were measured according to NEMA NU2-2012 protocol. The region of interest (ROI) was draw at the center of each hot sphere with a matched diameter to the sphere. The background ROI was drawn in the peripheral area of the phantom background at the central slice of the spheres, and $\pm$1 cm and $\pm$2 cm aside. A total of 60 background ROIs of each size, 12 ROIs on each of five slices shall thus be drawn. The locations of all ROIs shall be fixed between successive measurements. The average counts inn each background ROI shall be recorded. The percent contrast CR_H, j_ for each hot sphere j and the percent background variability BV_j_ are calculated by equation (4), (5) and (6):

$${CR}_{H,j}=\left( {C_{H,j}}/{C_{B,j}}-1 \right)/{\left( {a_{H}}/{a_{B}}-1 \right)\times100 \% \left( 4 \right)}$$

$${BV}_{j}={{SD}_{j}}/{C_{B,j}}\times100 \% \left( 5 \right)$$

${SD}_{j}=\sqrt{{\sum_{k=1}^{K} \left( C_{B,j,k}-C_{B,j} \right)^{2}}/\left( K-1 \right)} \left( 6 \right)$

Where *CR_H,j_* is the percent CR of the sphere *j*, *C_H,j_* and *C_B,j_* are the average counts within a ROI on each sphere *j* and corresponding background ROIs. *a_H_* and *a_B_* are the activity concentration in the sphere and the background of the phantom, *BV_j_* is the percent *BV* measured by background ROIs from sphere *j*, *SD_j_* is the standard deviation of the background ROI counts for sphere *j*. *K* is equal to 60.
